# A novel mutation in *FN1* causing spondylometaphyseal dysplasia corner fracture type in a multigenerational family

**DOI:** 10.1210/jcemcr/luag116

**Published:** 2026-04-22

**Authors:** Christopher Woody, Ronald Gwinn, Rose Guo, Aimee Morrow, Janet Rubin

**Affiliations:** Department of Medicine, University of North Carolina at Chapel Hill, Chapel Hill, NC 25799-7170, USA; Department of Medicine, University of North Carolina at Chapel Hill, Chapel Hill, NC 25799-7170, USA; Cone Health Pediatric Specialists Genetics, Greensboro, NC 27401, USA; Cone Health Pediatric Specialists Genetics, Greensboro, NC 27401, USA; Department of Medicine, University of North Carolina at Chapel Hill, Chapel Hill, NC 25799-7170, USA

**Keywords:** early osteoporosis, spondylometaphyseal dysplasia, corner fracture

## Abstract

We report a case of a female individual with severe spine osteoporosis diagnosed at age 52. History revealed a childhood diagnosis of spondylometaphyseal dysplasia corner fracture type (SMDCF), a rare skeletal dysplasia associated with short stature and scoliosis. Genetic analysis showed a novel heterozygous variant in the fibronectin 1 gene (*FN1*) which mapped to 4 affected family members. The site of the familial pathogenic variant (c.643T>C, p.Cys215Arg) was located in the part of the fibronectin protein associated with established pathogenic variants, which interfere with secretion of the protein from mesenchymal stem cells. Little has been reported regarding the natural history of SMDCF: our patient presented to the adult endocrine clinic with early postmenopausal osteoporosis. Her scoliosis was mild, and she admitted to continued limb pain that had been present since childhood.

## Introduction

Spondylometaphyseal dysplasias (SMD) are a group of rare disorders causing abnormal development of vertebrae and long bone metaphyses. SMD-corner fracture type (SMDCF) is a rare autosomal dominant variant of this skeletal dysplasia with distinctive radiographic features, including coxa vara, vertebral body abnormalities, short stature, and the irregular, corner-like appearance of the metaphyseal-diaphysis junction that gives the subtype its name. First described in 1990, SMDCF is associated with pathogenic variants in the fibronectin 1 (*FN1*) gene, a protein secreted by bone osteoprogenitor cells that is essential for extracellular matrix organization and bone formation. Typical variants disrupt normal bone development, leading to characteristic skeletal manifestations observed in affected individuals: in 2020, only 45 individuals had been described [1].

Pathogenic variants in *FN1* have been associated with a range of skeletal phenotypes, including spondylometaphyseal dysplasia and SMDCF in particular [2]. However, the mutational spectrum remains limited, and novel variants continue to provide critical insight into disease mechanisms. In this case report, we describe a previously unreported heterozygous *FN1* pathogenic variant in a family with clinical and radiographic features consistent with SMDCF. Our findings broaden the phenotypic and genotypic spectrum of FN1-related disorders.

## Case presentation

A 55-year-old woman was referred for care in our clinic for a diagnosis of osteoporosis; she was finishing a 2-year course of parathyroid hormone analogue therapy for treatment of severe osteoporosis. She endorsed limited mobility and chronic musculoskeletal pain. Birth history was unremarkable and there was no history of trauma. She did not report a history of fractures. She had had bilateral hip arthroplasties to improve coxa vara and gait abnormalities performed ∼8 years of age. She was found to have near-sighted visual impairment at approximately age 10 and began wearing corrected lenses at that time. Her vision continued to worsen through adulthood, eventually plateauing after menopause to an impairment of −7.5 diopters in the right eye and −8.75 diopters in the left eye. Menopause was at age 51 years. Previous biochemical workup revealed history of hypovitaminosis D treated with vitamin D of 5000 IU daily since. Physical examination revealed short stature (142 cm), waddling gait, loose teeth, short extremities with bowing of the arms, short trunk, scoliosis, coxa vara, and genu valgum, for which patient remembered wearing leg braces as child but does not recall workup for rachitic/osteomalacic disorders. We did not have childhood x-rays to assess typical corner fracture-like lesions typically seen in distal long bones but note that these tend to disappear after the growth plates fuse.

## Diagnostic assessment

**Figure 1:**
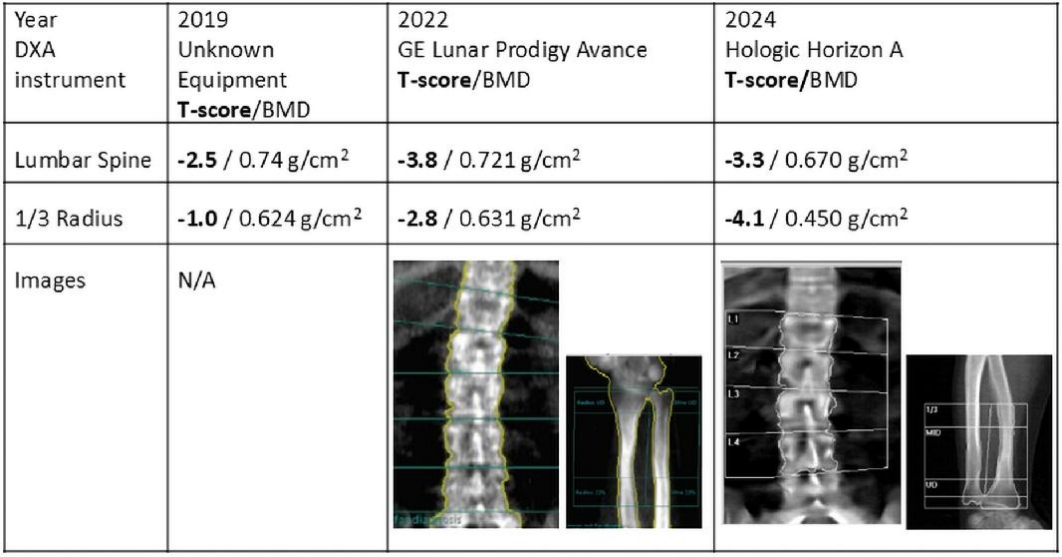
Patient densitometry.

**Figure 2:**
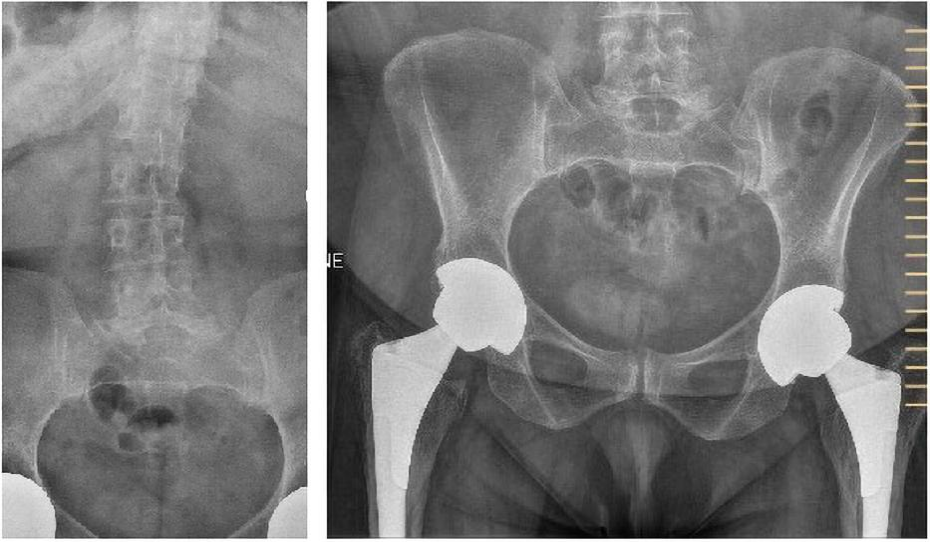
Skeletal findings age 52. Lumbar x-ray with diffuse osteopenia and mild scoliosis. Pelvis shows bilateral hip arthroplasty and diffuse osteopenia.

One year post menopause (age 52), densitometry of spine showed a T-score of −3.8, which had decreased in the preceding 3 years (Fig. 1). X-rays of the spine and pelvis after 2 years of parathyroid hormone analogue therapy demonstrated mild lumbar scoliosis (Cobb angle 12%) and diffuse osteopenia (Fig. 2). There was no evidence of thyroid dysfunction (thyroid stimulating hormone 1.350 µIU/mL) (normal reference range 0.550-4.780 µIU/mL), calcium was 9.6 mg/dL (SI: 2.395 mmol/L) (reference range, 8.5-10.2 mg/dL, [2.121-2.545 nmol/L]), phosphorous was 4.2 mg/dL (SI: 1.355 mmol/L) (reference range 2.4-5.1 mg/dL, [SI: 0.774-1.645 mmol/L)] and parathyroid hormone was 53.1 pg/mL (SI: 5.631 pmol/L) (reference range 18.4-80.1 pg/mL, [SI: 1.951-8.494 pmol/L]). Bone turnover markers were measured during the genetic inquiry, including 24-hour urine calcium, N-telopeptide, beta-crosslaps, and N-terminal pro-peptide type 1 procollagen, all of which were normal for a postmenopausal woman.

The patient's grandmother (deceased), mother (height 152 cm), brother (157.5 cm), and 1 of her 2 daughters (152 cm) reportedly had similar phenotype. Her unaffected daughter had a height of 167.6 cm. The patient's father was 185.4 cm and her husband was 195.6 cm. Patient provided documentation from her brother's treatment, which consisted of 4 separate surgeries for correction of bilateral coxa vara from 1969 to 1977. Per patient report, her mother had osteoporotic fractures of the spine and ankle after ground level falls in her late 70s. No consanguinity was noted.

**Figure 3:**
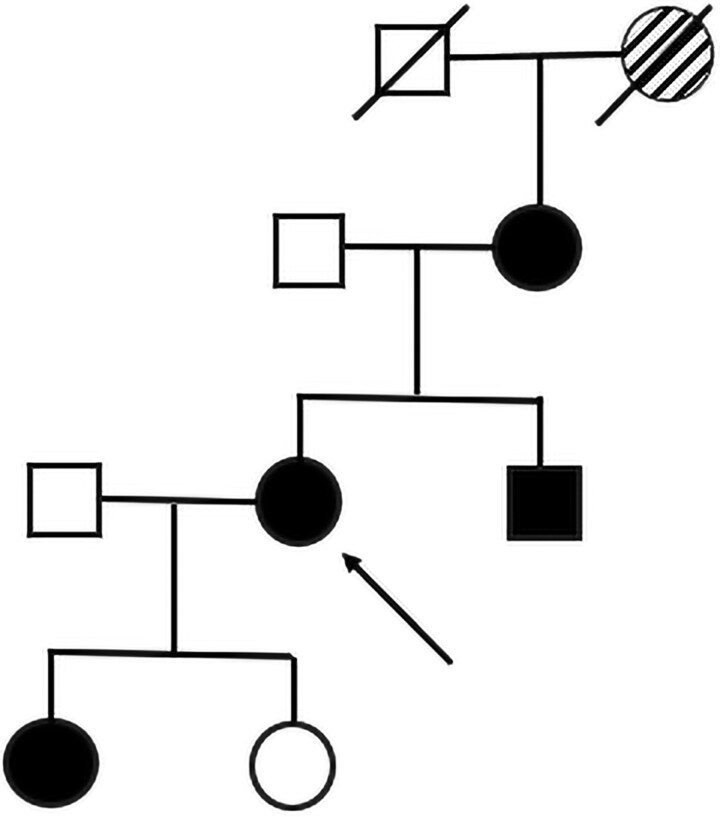
Pedigree analysis. The proband is indicated by the arrow. Circles indicate females; squares indicate males; black indicates affected phenotype and confirmed *FN1* variant; partially shaded indicates affected phenotype without confirmed *FN1* variant; slash through indicates deceased.

The patient underwent sequence analysis (Invitae Skeletal Disorders Panel) revealing a heterozygous missense variant in the *FN1* gene on chromosome 2, c.643T>C, p.Cys215Arg, reported as a variant of uncertain significance (VCV002837997.3—ClinVar—NCBI). Affected family members (mother, brother, and daughter) had genetic testing which showed the same variant (Fig. 3). The variant was absent in the patient's unaffected daughter. The patient's grandmother had the same phenotype as the affected individuals but was not genotyped.

## Treatment

After completing 2 years of parathyroid hormone analogue therapy, the patient was started on 60 mg every 6 months denosumab dosing with a follow-up density assessment planned in 2 years.

## Outcome and follow-up

The patient was seen in our clinic 1 year after beginning the current regimen of denosumab. She was agreeable to continuation of denosumab for 2-year total course with plans for future consolidation with zoledronic acid.

## Discussion

We present a patient referred for early-onset osteoporosis post menopause with a known history of short stature, genu valgum, and coxa vara status after bilateral hip arthroplasties, who had a family history significant for similar phenotype throughout multiple generations. Genetic testing showed that the patient and affected family members had a novel *FN1* variant c.643T>C, p.Cys215Arg, which, similar to known pathogenic variants, was within the N-terminus of the fibronectin protein. Given the multigenerational skeletal dysplasia phenotype that maps with this variant described in the pedigree above, we recommend that the c.643T>C, p.Cys215Arg variant present in 4 family members be classified as pathogenic for SMDCF.

Spondylometaphyseal dysplasias are rare skeletal disorders causing abnormalities of vertebrae and metaphyses of long bones. The most common subtype of SMD is Kozlowski type (SMDK), caused by a mutation in *TRPV4* leading to a gain-of-function mutation in the TRPV4 calcium channel, disrupting chondrocyte differentiation [3]. Phenotypically, SMDK is characterized by flattened vertebrae with kyphosis and metaphyseal abnormalities in the pelvis [4]. Spondylo-meta-epiphyseal dysplasia (SMED-SL) arises from a mutation in discoidin domain receptor 2 gene affecting collagen cross-linking and growth regulation signaling [5]. A mutation in glutathione peroxidase 4 (*GPX4*) underlies Sedaghatian-type spondylometaphyseal dysplasia (SSMD), a fatal autosomal recessive subtype consisting of fetal skeletal, central nervous system and cardiac abnormalities [6]. Another autosomal recessive subtype is Shwachman-Bodian-Diamond syndrome (SBDS), typified by mild/moderate short stature and ascribed to errors in RNA metabolism [7]. SMDCF, in turn, can be distinguished from these spondylometaphyseal dysplasias by the presence of asymmetrical microfractures in ossification centers in the metaphyses of long bones. These appear throughout childhood and adolescence and resolve in adulthood as the growth plates close. Aside from these distinct imaging findings, SMDCF is differentiated from the most common SMDK by the presence of scoliosis of differing severity with short stature and association with visual impairment [8], such as seen in our patient.

SMDCF has been linked to variants in the *COL2A1* type II collagen gene, which has been reported in a wide range of skeletal dysplasias, such as Stickler Syndrome, Kniest Dysplasia, and Spondyloepiphyseal Dysplasia Congenita [9]. More recently, SMDCF was shown to be associated with missense variants in fibronectin 1 [2, 10], which are clustered in the fibronectin type I (FNI) N-terminus domain [11]. The location of these variants is thought to disrupt the normal disulfide bond formation within the N-terminus domain between cysteine residues: such a disruption in fibroblasts is suggested to inhibit fibronectin secretion and the formation of cell matrix [10]. The decreased secretion of the mutated fibronectin, and the inability of the secreted protein to form proper fibrils, contributes to a dysregulated extracellular matrix, thus impairing ossification [2, 10].

Case series have demonstrated similar cysteine missense variants as that seen in our patient in a young population ranging in age from 6.8 years to 32 years, with subjects displaying the expected phenotype of short stature, coxa vara, and irregular metaphyses with corner fractures, as well as a large proportion with scoliosis [8]. These phenotypes arising from *FN1* mutations are copied in our patient, supporting that the novel missense cysteine residue variant reported here is pathogenic. Reporting suggests that patients with fibronectin 1-related SMDCF have severe scoliosis [1], as well as abnormalities in vertebrae; our patient had only moderate scoliosis and normal appearing vertebrae. Future investigations may be able to link the level of extracellular fibronectin to phenotype.

The natural history of SMDCF into later life has not been reported. Our patient's early osteoporosis might suggest that an association with fibronectin dysfunction, perhaps similar to the early osteoporosis of osteogenesis imperfecta due to abnormalities in the collagen matrix. As such, it may be reasonable to include genetic testing for SMDCF in individuals with early-onset osteoporosis and skeletal irregularities.

## Learning points

- This case highlights a novel autosomal dominant *FN1* variant causing SMDCF.
- Our findings expand the mutational spectrum of *FN1*-associated skeletal dysplasias and underscore the importance of genetic testing in atypical or familial cases of metaphyseal dysplasia.
- Skeletal dysplasias may come to attention in patients presenting with early osteoporosis.

## Data Availability

Data sharing is not applicable to this article as no datasets were generated or analyzed during the current study.

## Abbreviations

FN1: fibronectin type I
SMD: spondylometaphyseal dysplasia
SMDCF: spondylometaphyseal dysplasia corner fracture type
SMDK: spondylometaphyseal dysplasia Kozlowski type

## References

[1] England J, Campeau PM. Spondylometaphyseal dysplasia, corner fracture type. In: Adam MP, Bick S, Mirzaa GM, Pagon RA, Wallace SE, Amemiya A, eds. GeneReviews®. University of Washington; 1993:1993‐2026. 32200603

[2] Lee CS, Fu H, Baratang N, et al Baylor-Hopkins Center for Mendelian G, cohn DH, tartaglia M, lee BH, reinhardt DP, campeau PM. Mutations in fibronectin cause a subtype of spondylometaphyseal dysplasia with “corner fractures”. Am J Hum Genet. 2017;101(5):815‐823. 29100092 10.1016/j.ajhg.2017.09.019 PMC5673654

[3] Dicks AR, Maksaev GI, Harissa Z, et al Skeletal dysplasia-causing TRPV4 mutations suppress the hypertrophic differentiation of human iPSC-derived chondrocytes. Elife. 2023;12:71154. 10.7554/eLife.71154 PMC9949800 36810131

[4] Krakow D, Vriens J, Camacho N, et al Mutations in the gene encoding the calcium-permeable ion channel TRPV4 produce spondylometaphyseal dysplasia, Kozlowski type and metatropic dysplasia. Am J Hum Genet. 2009;84(3):307‐315. 19232556 10.1016/j.ajhg.2009.01.021 PMC2667978

[5] Al-Kindi A, Kizhakkedath P, Xu H, et al A novel mutation in DDR2 causing spondylo-meta-epiphyseal dysplasia with short limbs and abnormal calcifications (SMED-SL) results in defective intra-cellular trafficking. BMC Med Genet. 2014;15(1):42. 24725993 10.1186/1471-2350-15-42 PMC4001364

[6] Fedida A, Ben Harouch S, Kalfon L, et al Sedaghatian-type spondylometaphyseal dysplasia: whole exome sequencing in neonatal dry blood spots enabled identification of a novel variant in GPX4. Eur J Med Genet. 2020;63(11):104020. 32827718 10.1016/j.ejmg.2020.104020

[7] Nishimura G, Nakashima E, Hirose Y, et al The shwachman-bodian-diamond syndrome gene mutations cause a neonatal form of spondylometaphysial dysplasia (SMD) resembling SMD sedaghatian type. J Med Genet. 2007;44(4):e73. 17400792 10.1136/jmg.2006.043869 PMC2598034

[8] Costantini A, Valta H, Baratang NV, et al Novel fibronectin mutations and expansion of the phenotype in spondylometaphyseal dysplasia with “corner fractures”. Bone. 2019;121:163‐171. 30599297 10.1016/j.bone.2018.12.020

[9] Barat-Houari M, Dumont B, Fabre A, et al The expanding spectrum of COL2A1 gene variants IN 136 patients with a skeletal dysplasia phenotype. Eur J Hum Genet. 2016;24(7):992‐1000. 26626311 10.1038/ejhg.2015.250 PMC5070901

[10] Cadoff EB, Sheffer R, Wientroub S, Ovadia D, Meiner V, Schwarzbauer JE. Mechanistic insights into the cellular effects of a novel *FN1* variant associated with a spondylometaphyseal dysplasia. Clin Genet. 2018;94(5):429‐437. 30051459 10.1111/cge.13424 PMC6175647

[11] Dinesh NEH, Rousseau J, Mosher DF, et al Mutations in fibronectin dysregulate chondrogenesis in skeletal dysplasia. Cell Mol Life Sci. 2024;81(1):419. 39367925 10.1007/s00018-024-05444-4 PMC11456097

